# Fractional coalescent

**DOI:** 10.1073/pnas.1810239116

**Published:** 2019-03-13

**Authors:** Somayeh Mashayekhi, Peter Beerli

**Affiliations:** ^a^Department of Scientific Computing, Florida State University, Tallahassee, FL 32306

**Keywords:** coalescent, fractional calculus, population genetics, Bayesian inference, environmental heterogeneity

## Abstract

An approach to the coalescent, the fractional coalescent (f-coalescent), is introduced. The derivation is based on the discrete-time Cannings population model in which the variance of the number of offspring depends on the parameter α. This additional parameter α affects the variability of the patterns of the waiting times; values of α<1 lead to an increase of short time intervals, but occasionally allow for very long time intervals. When α=1, the f-coalescent and the Kingman’s n-coalescent are equivalent. The distribution of the time to the most recent common ancestor and the probability that n genes descend from m ancestral genes in a time interval of length T for the f-coalescent are derived. The f-coalescent has been implemented in the population genetic model inference software Migrate. Simulation studies suggest that it is possible to accurately estimate α values from data that were generated with known α values and that the f-coalescent can detect potential environmental heterogeneity within a population. Bayes factor comparisons of simulated data with α<1 and real data (H1N1 influenza and malaria parasites) showed an improved model fit of the f-coalescent over the n-coalescent. The development of the f-coalescent and its inclusion into the inference program Migrate facilitates testing for deviations from the n-coalescent.

## Significance

The fractional coalescent is a generalization of Kingman’s n-coalescent. It facilitates the development of the theory of population genetic processes that deviate from Poisson-distributed waiting times. It also marks the use of methods developed in fractional calculus in population genetics. The fractional coalescent is an extension of Canning’s model, where the variance of the number of offspring per parent is a random variable. The distribution of the number of offspring depends on a parameter α, which is a potential measure of the environmental heterogeneity that is commonly ignored in current inferences.

In 1982, Kingman (1, 2) introduced the n-coalescent. The n-coalescent describes the probability density function of a genealogy of samples embedded in a population with fixed size. Extensions to this probabilistic description of the genealogical process include changing population size (3, 4), immigration (5, 6), population divergence (7), selection (8), and recombination (9). These theoretical advances resulted in several widely used computer packages that estimate various population parameters (for example, refs. 10–12). While the waiting times for events in the n-coalescent are exponentially distributed, a more general framework of these waiting times is offered by the field of fractional calculus (13–18). Fractional calculus has attracted considerable interest because of the ability to model complex phenomena, such as continuum and statistical mechanics (19) and viscoelastic materials (20). We introduce fractional calculus into population genetics. Our work concentrates on the use of the fractional Poisson process (21) in the context of the coalescent, and we introduce a model of coalescence, the fractional coalescent, or f-coalescent. We derive the f-coalescent based on the discrete-time Cannings model and present the properties of the f-coalescent. This f-coalescent is then implemented in a Bayesian estimator of effective population size; we discuss the implementation and runtime characteristics. We explore the quality of the inference for simulated datasets and also apply the method to three real datasets: mitochondrial sequence data of humpback whales (22), mitochondrial data of the malaria parasite *Plasmodium falciparum* (23), and complete genome data of the H1N1 influenza virus strain collected in Mexico City in 2014. The biological motivation of this model is discussed by using a simulator that assigns an environmental quality affecting the chance of having offspring to each individual of a population. The dataset which is derived based on this simulator shows the potential heterogeneity within a population. It is shown that the f-coalescent is a better model than the n-coalescent to describe the variability of this dataset.

## Motivation

It is common to assume that, within a population, all individuals are affected in the same way by the environment (3–7, 9–12). Neglecting this heterogeneity may lead to biased parameter estimates. Development of multiple-merger coalescence focused on either strong selection (24) or large offspring variance (25); both could be induced by environmental heterogeneity. But, these approaches do not allow estimating a parameter that reflects this heterogeneity. The f-coalescent allows nonexponential waiting times; therefore, it should be able not only to handle datasets generated under such conditions, but also give estimates about the magnitude of this heterogeneity.

## Model

We derive the f-coalescent based on the nest-site model which was introduced by Wakeley (26). We included the derivation of the f-coalescent from the discrete Cannings model (*SI Appendix*, section B) and an alternative derivation of the f-coalescent as a semi-Markov process, in an equivalent way as the n-coalescent emerges as a continuous-time Markov process (*SI Appendix*, section C). Since we compare the f-coalescent with the Kingman’s n-coalescent, we have included a derivation of Kingman’s n-coalescent for the Wright–Fisher and the Cannings model in *SI Appendix*, section A.

### The *f*-Coalescent Based on the Nest-Site Model.

The nest-site model allows for different qualities of nest sites, therefore leading to differences among offspring numbers, leading to the Canning model. The habitat structure determines the distribution of offspring numbers. Consider a haploid population model with a fixed population size N. Individuals can occupy places with reproduction conditions 1,…,L. Consider N individuals per generation, where fixed proportions $\beta_{1},\ldots ,\beta_{L}\geq 0$ of them have condition i ($\sum_{i}\beta_{i}=1$) and the total number of offspring of all individuals in condition i is $N\chi_{i}$, where $\chi_{i}\in \left[0,1\right]$ fixed with $\sum_{i}\chi_{i}=1$. Assume the $N\chi_{i}$ offspring are produced by their $N\beta_{i}$ parents via Wright–Fisher sampling. In this model, $\beta_{i}$ and $\chi_{i}$ are fixed and constant across generations; therefore, Kingman’s coalescent, by changing the time scale to $N_{e}=N/\sigma^{2}$, is an appropriate model, where $\sigma^{2}=\sum_{i=1}^{L}\chi_{i}^{2}/\beta_{i}$ (details are in *SI Appendix*, section D).

If in this model the quality of nest sites is a random variable, then the probability of coalescence becomes a random variable and Kingman’s coalescent cannot be an appropriate model to describe this probability. Suppose $\chi_{i}$ is a discrete random variable, which is drawn once and is identical for each generation, whose possible values are $\chi_{i}^{j},\;j=1,2,\ldots$, where $\sum_{i}\chi_{i}^{j}=1,\;j=1,2,\ldots$. For each case, for example $\chi_{i}^{j}$, $N\chi_{i}^{j}$ offspring are produced by their $N\beta_{i}$ parents via Wright–Fisher sampling. Similar to ref. 26, the probability that two individuals come from the same parent in the immediately previous generation is

$$P\left\{\text{coal}|\chi_{1}=\chi_{1}^{j},\ldots ,\chi_{L}=\chi_{L}^{j}\right\}=\overset{L}{\underset{i=1}{\sum }}\chi_{i}^{j}\left(\frac{N\chi_{i}^{j}-1}{N}\right)\left(\frac{1}{N\beta_{i}}\right).$$ [1]

As N increases, the probability of coalescence, which is a random variable, becomes

$$P\left\{\text{coal}|\chi_{1}=\chi_{1}^{j},\ldots ,\chi_{L}=\chi_{L}^{j}\right\}\approx \frac{1}{N}\overset{L}{\underset{i=1}{\sum }}\frac{{\left(\chi_{i}^{j}\right)}^{2}}{\beta_{i}}.$$ [2]

This argument shows that, in each case j=1,2,…, with probability $\beta_{i}$, the individual will have a Poisson number of offspring with mean and variance equal to $\chi_{i}^{j}/\beta_{i}.$ Then, the expected number of its offspring is equal to 1. By conditioning on the type of nest site, the individual ends up occupying and the variance of the number of offspring then $\sigma^{2}$ becomes a random variable whose possible values are $\sigma_{j}^{2}$ with j=1,2,…, where

$$\sigma_{j}^{2}=\overset{L}{\underset{i=1}{\sum }}\beta_{i}\left(\frac{\chi_{i}^{j}}{\beta_{i}}+{\left[\frac{\chi_{i}^{j}}{\beta_{i}}\right]}^{2}\right)-1=\overset{L}{\underset{i=1}{\sum }}\frac{{\left(\chi_{i}^{j}\right)}^{2}}{\beta_{i}}.$$ [3]

Using Eqs. **2** and **3**, we have

$$N_{e}^{j}=\frac{N}{\sigma_{j}^{2}},$$ [4]

and

$$P\left\{\text{coal}|\chi_{1}=\chi_{1}^{j},\ldots ,\chi_{L}=\chi_{L}^{j}\right\}\approx \frac{1}{N_{e}^{j}}=\frac{\sigma_{j}^{2}}{N}.$$ [5]

Assume that the variance of the number of offspring is a random variable [$\sigma^{2}\in \left(0,\infty \right)$] which has the probability mass function $\omega \left(\sigma^{2},\alpha \right)$ where 0<α≤1. Suppose this probability mass function has a closed form which has been introduced in *SI Appendix*, Eq. **S60**; since 0<α≤1 is a parameter, $\omega \left(\sigma^{2},\alpha \right)$ can have different forms depending on the α. The relation between the probability mass function of $\chi_{i}$ and $\omega \left(\sigma^{2},\alpha \right)$ is presented in *SI Appendix*, section O.

By this assumption, similar to *SI Appendix*, Eq. **S5**, the probability that the two lineages remain distinct for N units of scaled time is

$$P\left\{\text{not coal}\;|\sigma^{2}=\sigma_{j}^{2}\right\}={\left(1-\frac{\sigma_{j}^{2}}{N}\right)}^{N\tau }.\;\sigma_{j}^{2}\in \left(0,\infty \right).$$ [6]

By using *SI Appendix*, Eq. **S62**, the average of Eq. **6** over the distribution of $\sigma^{2}\in \left(0,\infty \right)$ shows the probability that the two lineages remain distinct for N units of scaled time as

$$E_{\omega }\left({\left(1-\frac{\sigma_{j}^{2}}{N}\right)}^{N\tau }\right)=\underset{j}{\sum }\omega \left(\sigma_{j}^{2},\alpha \right){\left(1-\sigma_{j}^{2}\frac{1}{N}\right)}^{N\tau }\to E_{\alpha }\left(-\tau^{\alpha }\right),$$ [7]

as N goes to infinity, $E_{\alpha }\left(-\tau^{\alpha }\right)$ is the Mittag–Leffler function (*SI Appendix*, section N) (27). We choose the time scale as $\tau =t/\left(N^{1/\alpha }\right)$; thus, in the limit, the coalescence time for a pair of lineages is distributed as the fractional generalization of the exponential distribution (28). We can generalize the f-coalescent from two lineages to k lineages by changing τ→τk21/α. The probability that the two lineages among k lineages remain distinct for N units of scaled time is

∑jω(σj2,α)1−σj2k21αNNτ→Eα(−k2τα). [8]

Choosing the time scale as $\tau =t/\left(N^{1/\alpha }\right)$ keeps the parameter (population size) the same as the n-coalescent (*SI Appendix*, section B).

Based on Eq. **6**, each value of the random variable $\sigma_{j}^{2}$ leads to Kingman’s n-coalescent genealogy on a suitable timescale which is a bifurcating genealogy (*SI Appendix*, Eq. **S12**). Eq. **7** shows that the average of these bifurcating genealogies leads to the f-coalescent on a suitable timescale, which still is a bifurcating genealogy (*SI Appendix*, Eq. **S15**). An alternative derivation which characterizes the f-coalescent as a semi-Markov process (*SI Appendix*, section C) shows that the f-coalescent does not require multiple mergers, similar to the n-coalescent. These different derivations of the f-coalescent suggest that we have a versatile framework that maintains the strictly bifurcating property of the n-coalescent, but permits more variability in waiting times between coalescent events. Thus, this versatility may allow us to infer processes that change the waiting times—for example, selection—better. Currently, coalescent models that allow multiple mergers, such as the BS-coalescent (cf. 24), are used to discuss such forces. The f-coalescent may be a viable alternative.

### Properties of the *f*-Coalescent.

The n-coalescent has two steps: First, choose a pair to coalesce by using the concept of equivalence classes; second, pick a waiting time in which two lineages need to coalesce. For the f-coalescent, we changed the second step, resulting in a different time to the most recent common ancestor ($T_{\text{MRCA}}$) compared with the n-coalescent. We derive this new distribution of the $T_{\text{MRCA}}$ of the f-coalescent and compare it with the $T_{\text{MRCA}}$ of the n-coalescent. We also present the probability that n genes are descendants from m ancestral genes using the f-coalescent and compare these results with the n-coalescent. To do this, we extend the work of ref. 29 to the f-coalescent. In the following theorems, we use Eq. **7**. These lead to the probability density of waiting times of the f-coalescent

$$f\left(t\right)=t^{\alpha -1}\lambda E_{\alpha ,\alpha }\left(-\lambda t^{\alpha }\right).$$ [9]

For α=1, this is equivalent to an exponential distribution which is used for the n-coalescent.

### Theorem 1.

*Suppose* $f_{T_{i}}\left(t\right)=t^{\alpha_{i}-1}\lambda_{i}E_{\alpha_{i},\alpha_{i}}\left(-\lambda_{i}t^{\alpha_{i}}\right)$ *is the distribution of a waiting time in the* f-*coalescent*, *where* $T_{i},\;i=2,\ldots ,n$ *are the coalescent times and* λi=i2 *if* $\alpha_{1}=\alpha_{2}=\ldots =\alpha_{n}$, *then the distribution of* $T_{\text{MRCA}}=\sum_{i=2}^{n}T_{i}$ *is*

$$f_{T_{\text{MRCA}}}\left(t\right)=\overset{n}{\underset{i=2}{\sum }}\left(\overset{n}{\underset{\begin{array}{c} k=2\\ k\neq n \end{array}}{\prod }}\frac{\lambda_{k}}{\lambda_{k}-\lambda_{i}}\right)f_{T_{i}}\left(t\right),$$ [10]

*or*, *equivalently*, *this can be presented as*

$$f_{T_{\text{MRCA}}}\left(t\right)=\overset{n}{\underset{i=2}{\sum }}\left(\frac{\left(2i-1\right){\left(-1\right)}^{i}n_{\left[i\right]}}{n_{\left(i\right)}}\right)f_{T_{i}}\left(t\right),$$ [11]

*where* $n_{\left[i\right]}=n\left(n-1\right)\ldots \left(n-i+1\right)$, $n_{\left(i\right)}=n\left(n+1\right)\ldots \left(n+i-1\right)$.

### Proof:

For the proof, see *SI Appendix*, section E.

In the next theorem, we derive the probability that n genes are descendants from m ancestral genes in the f-coalescent.

### Theorem 2.

*With the same assumption in Theorem* 1, *in the* f-*coalescent*, *the probability* $P_{nm}\left(T\right)$ *that* n *genes descended from* m *genes* T *units of time ago is*

$$P_{nm}\left(T\right)=\left\{\begin{array}{cc} \overset{n}{\underset{i=m+1}{\sum }}(\overset{n}{\underset{\begin{array}{c} k=m+1\\ k\neq n \end{array}}{\prod }}\frac{\lambda_{k}}{\lambda_{k}-\lambda_{i}}(\left(\frac{\lambda_{i}}{\lambda_{m}-\lambda_{i}}E_{\alpha }\left(-\lambda_{i}T^{\alpha }\right)\right.\\ \;+\frac{\lambda_{i}}{\lambda_{i}-\lambda_{m}}E_{\alpha }\left(-\lambda_{m}T^{\alpha }\right)) & 1<m<n\\ \overset{n}{\underset{i=2}{\sum }}(\overset{n}{\underset{\begin{array}{c} k=2\\ k\neq n \end{array}}{\prod }}\frac{\lambda_{k}}{\lambda_{k}-\lambda_{i}})\left(-E_{\alpha }\left(-\lambda_{i}T^{\alpha }\right)+E_{\alpha }\left(0\right)\right) & m=1\\ E_{\alpha }\left(-\lambda_{n}T^{\alpha }\right) & m=n. \end{array}\right.$$ [12]

### Proof:

For the proof, see *SI Appendix*, section E.

### Corollary.

*The parameter* α *in Theorem* 1 *affects the variability in the patterns of the waiting times which*, *as a result*, *affects the distribution of time to the most recent common ancestor*, $f_{T_{\text{MRCA}}}\left(t\right)$. *While for the* n-*coalescent*, *as the sample size increases*, *the distribution of* $f_{T_{\text{MRCA}}}\left(t\right)$ *converges on a distribution with mean equal* 2, *which corresponds to a period of* 2N *generations in the haploid Wright*–*Fisher model*, *for the* f-*coalescent*, *we have a heavy*-*tailed distributions as the sample size increases*. *By using* *Eq.* **12**, *in the* f-*coalescent*, *it is more likely that* n *genes sampled from a population have more ancestors in a unit time compared with the* n-*coalescent*. *We give some numerical value of* *Eq.* **12** *for different values of* α *in* *SI Appendix*, *section F*. *In the* n-*coalescent* (α=1) m *decreases quite rapidly as* T *increases*, *but this is not the case in the* f-*coalescent* (*SI Appendix*, *section F*).

More details related to these two theorems are presented in *SI Appendix*, section E. We also derive the time to the most recent common ancestor in Methods and Results empirically, to compare the f-coalescent with the Bolthausen–Sznitman-coalescent (BS-coalescent).

### Probability Density Function of a Genealogy Based on the *f*-Coalescent.

To extract a particular genealogy G out of the many possible topologies defined by the interval times $u_{2},u_{3},\ldots ,u_{K}$, we need to take into account the number of possible configurations at any time $u_{k}$; by using Kingman’s n-coalescent for any $u_{k}$ and k lineages, there are k2 potential configurations, and we pick one particular one. If we use the same assumption for f-coalescent that only two lineages per generation can coalesce, then we get:

f(G|Θ)=∏k=2Kukα−1k(k−1)ΘEα,α(−λkukα)1k2, [13]

$$=\overset{K}{\underset{k=2}{\prod }}\left(u_{k}^{\alpha -1}\left[\frac{2}{\Theta }\right]E_{\alpha ,\alpha }\left(-\lambda_{k}u_{k}^{\alpha }\right)\right),$$ [14]

where K is the number of samples and Θ is the mutation-scaled population size (details are in *SI Appendix*, section H).

### Implementation.

We implemented our model in the existing framework of the software Migrate (10). In this framework, we approximate the Bayesian posterior density f(ρ|X,α)=f(ρ)f(X|ρ,α)/f(X|α), where X is the data and ρ is the parameter set for a particular population model—here, it is the mutation-scaled population size Θ—and α is a fixed parameter for the Mittag–Leffler function. The software uses Markov chain Monte Carlo (MCMC) to approximate the posterior density, calculating f(ρ) and f(X|ρ,α). To choose a tree genealogy during the MCMC, we draw new times for events. Details of the tree-changing algorithm are described by ref. 30. To draw a new time ($t_{0}$), we solve

$$P\left(t>t_{0}\right)=1-\int_{0}^{t_{0}}u^{\alpha -1}\lambda_{k}E_{\alpha ,\alpha }\left(-\lambda_{k}u^{\alpha }\right)du=E_{\alpha }\left(-\lambda_{k}t_{0}^{\alpha }\right),$$ [15]

where k and α are fixed numbers, and we choose random numbers between (0,1) for $P\left(t>t_{0}\right)$.

Using Eq. **15** to draw times directly is time-consuming. Therefore, we use the sampling method of the Mittag–Leffler distribution which has been introduced by MacNamara et al. (28). Since the Mittag–Leffler function can be expressed as a mixture of exponential functions, the fast simulation of geometric stable distributions can be used to sample the time. As a result, the time derived from the Mittag–Leffler function is

$$t_{0}=-{\left(\frac{1}{\lambda_{k}}\right)}^{\frac{1}{\alpha }}{\left(\frac{sin\left(\pi \alpha \right)}{tan\left(\pi \alpha \left(1-r_{1}\right)\right)}-cos\left(\pi \alpha \right)\right)}^{\frac{1}{\alpha }}log\left(r_{2}\right),$$ [16]

where $r_{1}$ and $r_{2}$ are two independent random numbers. More details for Eq. **16** have been presented by MacNamara et al. (28). The details related to the implementation of the Mittag–Leffler function are shown in *SI Appendix*, section I (31).

## Methods and Results

### Time to the Most Recent Common Ancestor for the f-Coalescent.

**Fig. 1.**
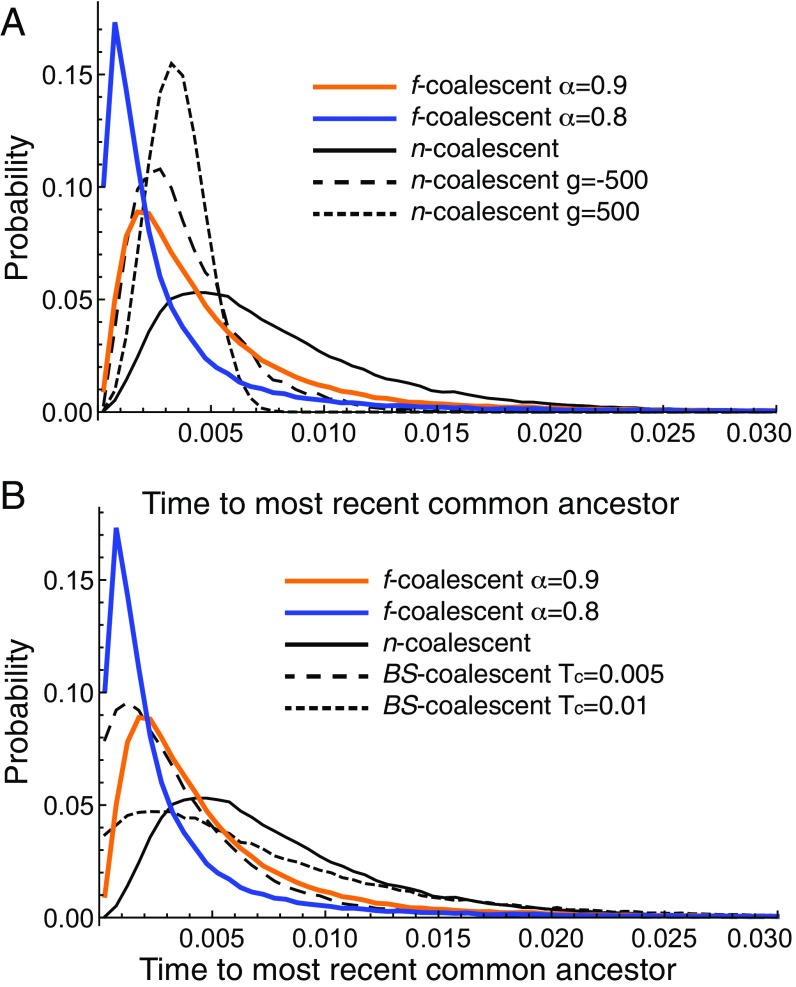
Empirical distribution of the time of the most recent common ancestor for various coalescents: strictly bifurcating (*A*) and f-coalescent vs. n-coalescent and multifurcating BS-coalescent (*B*). The *x* axis is truncated at 0.03. Each curve represent a histogram of 100,000 draws of the $T_{\text{MRCA}}$.

We compared empirical distributions of the $T_{\text{MRCA}}$ for a sample of five individuals for the f-, n-, and BS-coalescent (32) (more details are in *SI Appendix*, section J) (33). Fig. 1 shows examples of empirical distributions of the $T_{\text{MRCA}}$ for a sample of five individuals for the n-coalescent, the f-coalescent with two different values for α and the BS-coalescent. Each curve is based on 100,000 simulated $T_{\text{MRCA}}$ values. With α<1, the distribution becomes more peaked with more short time intervals and rare large time intervals, leading to heavier tails than with the n-coalescent; median values for the $T_{\text{MRCA}}$ of the different models were 0.00379 for the f-coalescent with α=0.9 and 0.00026 with α=0.8; 0.00667, 0.00343, and 0.00031 for the n-coalescent with no growth, strong growth, and strong shrinkage, respectively; the BS-coalescent had medians of 0.00576 with $T_{C}=0.01$ and 0.00288 with $T_{C}=0.005$. The expectation of the $T_{\text{MRCA}}$ for the n-coalescent for five samples simulated with a Θ=0.01 is 0.008. The expected $T_{\text{MRCA}}$ for the f-coalescent is infinite because of the heavy tail (cf. 34). Comparisons with the BS-coalescent are more difficult because of the parametrization (details are in *SI Appendix*, section J). We recognize that distributions of some the f-coalescent$T_{\text{MRCA}}$ and some of the BS-coalescent$T_{\text{MRCA}}$ look rather similar compared with the others, but the mapping of the parameter $T_{c}$ and Θ and comparison of the f-coalescent with the BS-coalescent will need further investigation.

### Simulation.

**Fig. 2.**
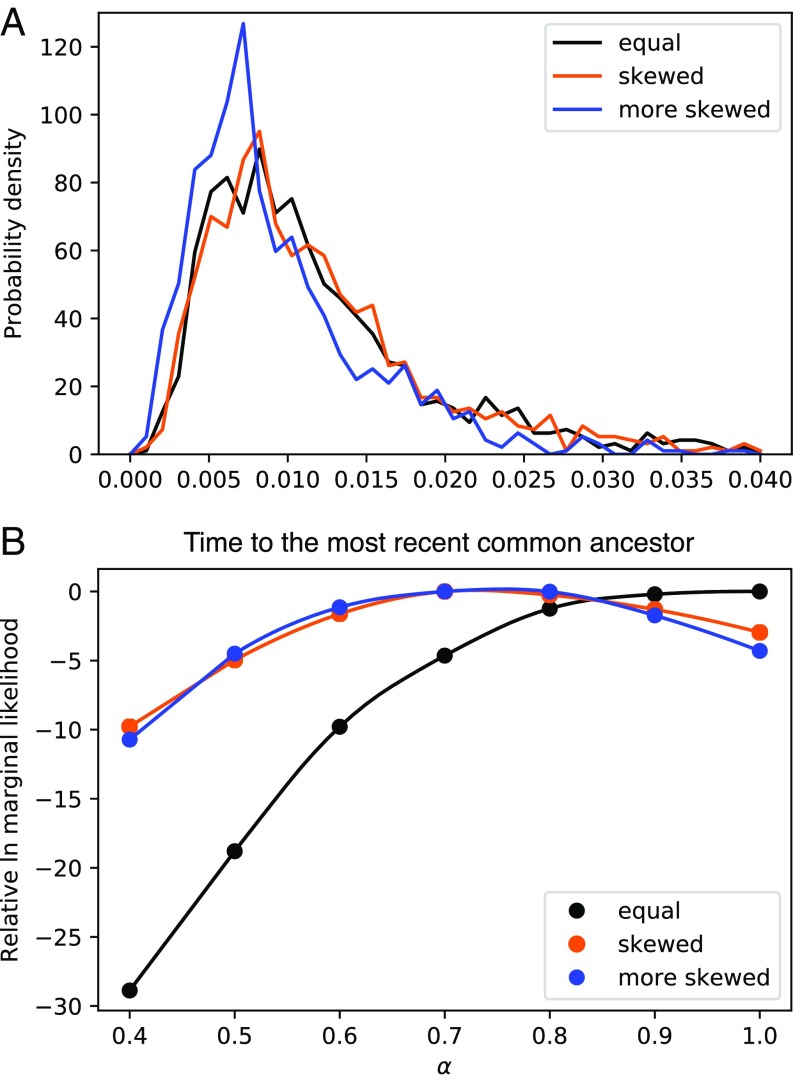
Comparison of the effect of variable environment on genealogy and estimation. (*A*) Effect on the time of the most recent common ancestor; histogram of 1,000 independent genealogies of 10 individuals. (*B*) Ln mL of a single-locus dataset for different values of α as a measure of the effect of the environment on the data.

We evaluated the algorithms using simulations. We updated our simulator package (available at https://github.com/pbeerli/fractional-coalescent-material) to allow generating genealogies from the f-coalescent (details are in *SI Appendix*, section J) (35–38). In general, it will be difficult to recover the parameter α that was used to simulate the data (*SI Appendix*, Fig. 2); data simulated with a particular α have a considerable range when estimated. A problem with these simulations is that the number of variable sites is dependent on the mutation-scaled population size Θ and the simulated branch length. Despite having the same Θ=0.01 among all simulations, the number of variable sites varies considerably over the range of α: With an α=0.4, $∼\frac{78}{10,000}$ sites (mean) are variable, compared with α=1.0 with $\frac{253}{10,000}$ sites. With α≤0.4, it is common to see no variable sites in a locus; median among 100 loci is zero. Low-variability datasets are difficult to use in any coalescent framework.

### Simulated Heterogeneity and the f-Coalescent.

Environmental heterogeneity can have an effect on the capacity to produce offspring. We have developed a simple forward population simulator (open source software on https://github.com/pbeerli/fractional-coalescent-material) that assigns an environmental quality affecting the chance of each individual of having offspring; otherwise, the simulator assumes a Wright–Fisher population with constant population size and every generation the parental population dies. If the simulator runs long enough, then one can generate a population genealogy out of which we can draw a coalescent sample of n individuals. Using these genealogies, we then generated DNA-sequence datasets that come from different regimes: (*i*) the environmental quality is the same for all (“equal”); (*ii*) the environmental quality is different, with three categories with relative rates of 1, 2, and 4 (“skewed”); and (*iii*) with relatives rates of 1, 2, and 8 (“more skewed”). Fig. 2*A* shows the distribution of the $T_{\text{MRCA}}$ for the three scenarios. The results suggest that variable environments change the $T_{\text{MRCA}}$ to become more recent than those in invariant environments on which the standard coalescent is based. We compared the *f*-coalescent with our heterogeneity simulations, and Fig. 2*B* shows that the *f*-coalescent with α<1 may give good descriptions of the fluctuating environment in the past when we use Bayesian-model selection criteria.

### Comparing the f-Coalescent with Structured Coalescence and Population Growth.

The f-coalescent has an additional parameter α which could reflect hidden structure. To explore whether the parameter α responds to population structure, we simulated data using the structured coalescent for two populations exchanging migrants using three different magnitudes of gene flow (details are in *SI Appendix*, section K). These datasets were evaluated under two different scenarios: For scenario A, we sampled data from both subpopulations, and for scenario B, we collected data only from subpopulation 1. For scenario A, we ran models that assumed that the population is (*i*) not structured with α<1, (*ii*) the standard single-population n-coalescent, and (*iii*) a structured n-coalescent model. A Bayesian-model selection approach excludes all models with α<1. The nonstructured standard n-coalescent model is rejected for the low and medium gene-flow scenarios, but the single-population n-coalescent model is the best model for data simulated with high immigration rates (details are in *SI Appendix*, section K). For scenario B, we ran models that assume that the population is (*i*) not structured with α<1, (*ii*) the standard single-population n-coalescent, and (*iii*) a model that assumes a ghost population (39). Models that include high to very high immigration picked the n-coalescent model as the best, rejecting the f-coalescent and the ghost-population model. With low immigration rates, the f-coalescent model had higher marginal likelihoods (mLs) at α=0.9, suggesting that rare migrants will disturb the exponential waiting time pattern of a single n-coalescent population (*SI Appendix*, Tables S2 and S3).

### Real Data Results.

**Fig. 3.**
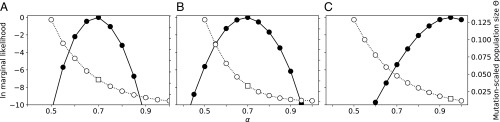
Model selection using relative mLs of an H1N1 influenza eight-locus (*A*), a *P. falciparum* mtDNA (*B*), and a humpback whale mtDNA (*C*) dataset. The solid line connects the ln mLs of models with different α values; the dashed line marks the mutation-scaled effective population size Θ; and the square marks Θ of the best model for each dataset.

We used three biological datasets to explore whether the f-coalescent could be a better fit than Kingman’s n-coalescent: an H1N1 influenza dataset of the Mexico City outbreak in 2014, a malaria parasite (*P. falciparum*), and a dataset of North Atlantic humpback whales (Fig. 3; detailed description is in *SI Appendix*, section J) (40–42). For the humpback whale data, model selection using mL suggests that models within the range of 0.8<α≤1.0 are preferred with relative log mL (relative lmL) >−2.07, which translates to model probabilities of >0.04; models with an α<0.6 had a model probability of 0.0000. The maximum mL was at α=0.95. The estimated mutation-scaled population size, Θ, varied considerably in this range. At α=0.8, Θ was 0.03030, and at α=1.0, Θ was 0.01170. The best model had a mode of Θ=0.01470 and a 95%−credibility for Θ from 0.0000 to 0.03480. Kingman’s n-coalescent is a good fit for the humpback whale dataset. For the malaria-parasite data, models within the range of 0.55<α≤0.85 had relative lmL >−3.49; all tested models within the range of 0.55<α<0.85 had model probabilities >0.01. The maximum mL was at α=0.7. The estimated mutation-scaled population size, Θ, varied considerably in this range. At α=0.55, Θ was 0.11007, and at α=0.85, Θ was 0.00693. The best model had a mode for Θ=0.03051 and a 95%−credibility interval from 0.02340 to 0.03762. Kingman’s n-coalescent was a poor fit the malaria-parasite data. The eight-segment dataset of the H1N1 strain of influenza from Mexico in 2014 had a well-defined maximum mL at α=0.7. Models within the range of 0.60≤α≤0.80 had relative lmL >−3.20; all tested models within the range of 0.55<α<0.85 had model probabilities > .01. The estimated mutation-scaled population size, Θ, varied considerably in this range. At α=0.6, Θ was 0.10530, and at α=0.80, Θ was 0.03210. The best model had a mode for Θ=0.05790 and a 95%−credibility interval from 0.02640 to 0.09504. Kingman’s n-coalescent was a poor fit the influenza data. We also ran a model that used the n-coalescent and exponential growth, estimating two parameters (growth g and Θ). The mL for the θ+G model (lmL = −19,455.27) was lower than the best model with α=0.80 (lmL = −19,338.28) and also lower than the constant-size n-coalescent model (lmL = −19,342.24); the relative lmL comparison with the best model was −118.14, suggesting that the growth model is inferior to the f-coalescent. Migrate output files are available in the github repository (https://github.com/pbeerli/fractional-coalescent-material).

## Discussion

A feature of the f-coalescent is the ability to accommodate very variable time intervals. Mixtures of very short branch lengths with very large branch lengths are possible, whereas the n-coalescent forces a more even distribution of these time intervals. Extensions of the n-coalescent to allow for population growth or population structure do not match the variability of time intervals of the f-coalescent. With exponential population growth, time intervals near the sampling date are enlarged, and near the root of the genealogy, the time intervals are shortened; the n-coalescents with exponentially shrinking populations also have heavy tails, but seem to have more longer branches than the f-coalescent. In the f-coalescent, time intervals near the tips are shortened, and time intervals near the root are lengthened. Analyses of data that were simulated by using a structured n-coalescent model show that only when we remove half of the simulated data and analyze a single subpopulation with models that assume that this is an isolated panmictic population will we get a better model fit with a f-coalescent model when the immigration rate is 1 per 10 generations. The unique mix of short and long waiting times of the f-coalescent thus may allow inferences with unknown compartmentalization that may mimic environmental heterogeneity within a single population, but we will need to extend our single-population f-coalescent to structured populations to study these types of models. The three real data examples suggest that the f-coalescent is a better fit to the data for the pathogens and not for the long-lived humpback whales. The mL comparison of different α for the humpback whales did not reject the Kingman’s n-coalescent, but the malaria-parasite data and the influenza data rejected the n-coalescent clearly. This may indicate that the f-coalescent may improve our understanding of evolution of long-lived vs. short-lived organisms or fast-evolving organisms that are under selection. The environmental heterogeneity within a population in these three datasets could be explained by the f-coalescent. The three datasets represent very different life-history strategies: Humpback whales live a long time, can move very far, and have only few offspring; the malaria parasite needs to be able to live in the saliva of mosquitoes and the bloodstream of vertebrates; and influenza viruses may encounter individual immune systems that may lead to high variability in the resulting dataset. Results show that environmental heterogeneity may have little effect on humpback whales, whereas the malaria-parasite and influenza data suggest that heterogeneity may need to be considered if we want to make informed decisions. The estimates of the effective population sizes and thus the diversity estimates of the different species is highly dependent on the heterogeneity parameter α—for example, the population size of influenza is considerably underestimated when using Kingman’s n-coalescent. Our introduction of the f-coalescent opens a window into further research that allows handling of heterogeneity that cannot be explained by population growth or population structure.

## Supplementary Material

Supplementary File
