# Supplementary material for: Fractional coalescent
Source: Proc Natl Acad Sci U S A. 2019 Mar 13;116(13):6244–9. doi: 10.1073/pnas.1810239116 (PMC6442577; doi:10.1073/pnas.1810239116)
Supplement: Supplementary File [file pnas.1810239116.sapp.pdf]

1. Supporting Information (SI)

A. The Kingman coalescent in the Wright-Fisher model and the Cannings model. In the Wright-Fisher model, the number of offspring of individuals in one generation is given by a multinomial distribution (1),

$$P(Y_1 = n_1, ..., Y_N = n_N) = \frac{N!}{n_1!...n_N!}\frac{1}{N^N} \quad \text{[SI1]}$$

where N is the population size and $Y_i, i = 1, ..., N$ is the number of offspring for each individual. Looking backward in time, in a haploid Wright-Fisher population of size N, the probability that two randomly selected individuals have the same parent in the previous generation is $\frac{1}{N}$, and the probability that they have different parents is $1 - \frac{1}{N}$. The probability that they do share a common parent after t generations is $(1 - \frac{1}{N})^t$. By using a suitable timescale τ such that one unit of scaled time corresponds to N generations, the probability that the two lineages remain distinct for N units of scaled time is

$$(1 - \frac{1}{N})^{N\tau} \to e^{-\tau}, \quad \text{[SI2]}$$

as N goes to infinity. Thus, in the limit, the coalescence time for a pair of lineages is exponentially distributed with mean 1 (2). The n-coalescent generalized the two lineages framework to k lineages by changing $\tau \to \tau\binom{k}{2}$, so the probability that the two lineages among k lineages remain distinct for N units of scaled time is

$$(1 - \frac{\binom{k}{2}}{N})^{N\tau} \to e^{-\binom{k}{2}\tau}, \quad \text{[SI3]}$$

as N goes to infinity. When we express $\tau = t/N$ then we recognize the familiar n-coalescent formula.
The Wright-Fisher model can be generalized. For example, it can be to extended to the Cannings model. Kingman showed that the coalescent holds for a subset of the exchangeable-type population models of Cannings in the limit as N tends to infinity when the time is appropriately rescaled. In the Cannings model, the number of offspring of all individuals (Y_i) has the same variance (σ^2). This variance changes the time scale to $N_e = \frac{N}{\sigma^2}$ in which

$$\lim_{N\to\infty} Var(Y_i) \to \sigma^2 \quad (3). \quad \text{[SI4]}$$

In this case, the probability that the two lineages remain distinct in the previous generation is

$$(1 - \frac{\sigma^2}{N}) = (1 - \frac{1}{N_e}). \quad \text{[SI5]}$$

The probability that the two lineages share a common parent after t generations is

$$(1 - \frac{1}{N_e})^t. \quad \text{[SI6]}$$

By using a suitable timescale τ such that one unit of scaled time corresponds to N_e generations, the probability that the two lineages remain distinct for N_e units of scaled time is

$$(1 - \frac{1}{N_e})^{N_e\tau} \to e^{-\tau}, \quad \text{[SI7]}$$

as N_e goes to infinity. Similar to Eq. (SI3), the probability that the two lineages among k lineages remain distinct for N_e units of scaled time is

$$(1 - \frac{\binom{k}{2}}{N_e})^{N_e\tau} \to e^{-\binom{k}{2}\tau}, \quad \text{[SI8]}$$

as N_e goes to infinity. When we express $\tau = t/N_e$ then we recognize the familiar n-coalescent formula.

B. The fractional coalescent based on the Cannings model. We use the Cannings model to derive the f-coalescent where the variance of the number of offspring depends on the parameter α. We use two different avenues: first, using the Cannings model directly; second, using the nest-site model. The second method is introduced in the main text 2A.

Der *et al.*(4) developed a generalized population model that includes the Cannings model as a special case, thus one could consider our f-coalescent also a special case. Our backward-time derivation is different from their derivation that is based on a forward-time process.

To derive the f-coalescent from the Cannings model directly, suppose that the variance of the number of offspring of all individuals depends on the parameter α as

$$\sigma^2 = \frac{1}{\Gamma(\alpha + 1)}, \quad \text{[SI9]}$$

www.pnas.org/cgi/doi/10.1073/pnas.1810239116
DRAFT

using Eqs. (SI5) and (SI9), the probability that the two lineages remain distinct in the previous generation is

$$(1-\frac{1}{\Gamma(\alpha+1)N}). \qquad \text{[SI10]}$$

The probability that they do share a common parents after t^{α} generation is $(1-\frac{1}{\Gamma(\alpha+1)N})^{t^{\alpha}}$. By using the suitable timescale τ^{α} such that one unit of scaled time corresponds to N generations, the probability that the two lineages remain distinct for N unit of scale time is

$$(1-\frac{1}{\Gamma(\alpha+1)N})^{N\tau^{\alpha}} \to e^{-\frac{\tau^{\alpha}}{\Gamma(1+\alpha)}} \to \mathcal{E}_{\alpha}(-\tau^{\alpha}), \quad 0<\tau<<1 \qquad \text{[SI11]}$$

as N goes to infinity. $\mathcal{E}_{\alpha}(-\tau^{\alpha})$ is the Mittag-Leffler function (SI:N). Thus, in the limit, the coalescence time for a pair of lineages is distributed as a Mittag-Leffler distribution. The n-coalescent generalized the two lineages framework to k lineages by changing $\tau \to \tau\binom{k}{2}$. Similarly we can generalized the f-coalescent from a two-lineages framework to k lineages by changing $\tau^{\alpha} \to \tau^{\alpha}\binom{k}{2}$. In the fractional coalescent we evaluate two separate parameters N and α while in Cannings model we have one parameter which we call the effective population size (N_e). Eq. (SI11) shows in the limit, the coalescence time for a pair of lineages is exponentially distributed with mean $1/\Gamma(\alpha+1)$ and we assume that there are no multiple mergers equivalent to the n-coalescent. The disadvantage of this derivation is related to τ which needs to be close to zero. To void this limitation, we derive the fractional coalescent based on the nest sites model in section 2A.

In this section and 2A we choose the same timescale. In both derivations, we assume the time scale as $\tau=\frac{t}{N^{\frac{1}{\alpha}}}$. We have two different choices for the time scale: first, $\tau=\frac{t}{N}$ which is similar to the time scale that we have in n-coalescent and second, $\tau=\frac{t}{N^{\frac{1}{\alpha}}}$. By choosing $\tau=\frac{t}{N}$ and plugging this time scale in Eqs. (8) and (SI11) the probability that two lineages remain distinct for N unit of scaled time is $\mathcal{E}_{\alpha}(-\tau^{\alpha})=\mathcal{E}_{\alpha}(\frac{-t^{\alpha}}{N^{\alpha}})$. If we call $t^{\alpha}=t$ then the probability leads to $\mathcal{E}_{\alpha}(\frac{-t}{N^{\alpha}})$. By choosing $\tau=\frac{t}{N^{\frac{1}{\alpha}}}$ and plugging this time scale in Eqs. (8) and (SI11) the probability that two lineages remain distinct for N unit of scaled time is $\mathcal{E}_{\alpha}(-\tau^{\alpha})=\mathcal{E}_{\alpha}(\frac{-t^{\alpha}}{N})$. If we call $t^{\alpha}=t$ then the probability leads to $\mathcal{E}_{\alpha}(\frac{-t}{N})$. In the second case we have the same interpretation of population size N as n-coalescent. Using the time scale $\tau=\frac{t}{N}$ in f-coalescent leads to have $\theta=2N^{\alpha}\mu$ as a parameter. Since in n-coalescent we have $\theta=2N\mu$ as a parameter, we have chosen the time scale $\tau=\frac{t}{N^{\frac{1}{\alpha}}}$ in f-coalescent to have the same parameter ($\theta=2N\mu$) as n-coalescent.

C. *Fractional* coalescent as a semi-Markov process. We can, also, describe the n-coalescent as a Markov process. We introduce a stochastic matrix P_N which is a single-step transition matrix as

$$P_N = I + \frac{Q}{N} + O(N^{-2}), \quad N \to \infty, \qquad \text{[SI12]}$$

where Q is the rate matrix of a Markov process. With an appropriate time scale proportional to N, after t generation we have

$$P_N^t = P_N^{\tau N} = (I+\frac{Q}{N})^{\tau N} \to e^{\tau Q}, \qquad \text{[SI13]}$$

as N goes to infinity. Eq. (SI13) is similar to Eq. (SI3) but in matrix form.

Using Eq. (9), we can find the following form of a Mittag-Leffler matrix function

$$\mathcal{E}_{\alpha}(\mathbb{A}t^{\alpha}) = \sum_i \omega(\sigma_i^2,\alpha)(I+\frac{-\sigma_i^2(-\mathbb{A})^{\frac{1}{\alpha}}}{N})^{N\tau}, \qquad \text{[SI14]}$$

where $\mathcal{E}_{\alpha}(\mathbb{A}t^{\alpha})$ is a stochastic matrix. Matrix $\mathbb{A}$ has two properties: Off-diagonal entries are positive or zero ($a_{ij}\geq 0, \quad i\neq j$) and diagonal entries ensure rows sum are zero ($a_{ii}=-\sum_{j,j\neq i} a_{ij}$). In the discrete case, the variance of the number of offspring can be a random variable, σ^2. For all σ_i^2 we need to introduce a single-step transition matrix. The rate matrix Q has the same properties as matrix $\mathbb{A}$. In Eq. (SI12), we substitute Q with $-\sigma_i^2(-Q)^{\frac{1}{\alpha}}$ and rewrite P_N as

$$P_N = I + \frac{-\sigma_i^2(-Q)^{\frac{1}{\alpha}}}{N}, \qquad \text{[SI15]}$$

and by introducing the appropriate time scale which is proportional to N, after t^{α} generation we have

$$P_N^t = P_N^{\tau N} = \sum_i \omega(\sigma_i^2,\alpha)(I+\frac{-\sigma_i^2(-Q)^{\frac{1}{\alpha}}}{N})^{N\tau} \to \mathcal{E}_{\alpha}(Q\tau^{\alpha}), \qquad \text{[SI16]}$$

Eq. (SI16) is similar to Eq. (9) but in matrix form. We can interpret $\mathcal{E}_{\alpha}(Q\tau^{\alpha})$ as a weighted sum of related Markov processes which itself is a semi-Markov process (SI:N).

DRAFT

D. Nest sites model. Nest-site model has been introduced in (3), which leads to the Cannings model. In this model, the habitat structure determines the distribution of offspring numbers. Consider a haploid population model with fixed population size N. Individuals can occupy places with reproduction conditions $1, ..., L$. Consider the N individuals per generation where fixed proportions $\beta_1, ..., \beta_L \geq 0$ of them have condition i ($\sum_i \beta_i = 1$) and the total number of offspring of all individuals in condition i is $\chi_i N$, where $\chi_i \in [0,1]$ is fixed with $\sum_i \chi_i = 1$. Assume the $N\chi_i$ offspring are produced by their $N\beta_i$ parents via Wright-Fisher sampling.
Consider the ancestry of a sample of size 2 under this model. The probability that the two individuals come from the same parent in the immediately previous generation is given by

$$P\{\text{coal}\} = \sum_{i=1}^{L} \chi_i (\frac{N\chi_i - 1}{N})(\frac{1}{N\beta_i}). \qquad \text{[SI17]}$$

This is the probability that both samples, taken without replacement, came from the part of the population that was produced by individuals in type i nest sites times the chance that they had the same parent given this. As N increases, the probability of coalescence becomes

$$P\{\text{coal}\} \approx \frac{1}{N} \sum_{i=1}^{L} \frac{(\chi_i)^2}{\beta_i}. \qquad \text{[SI18]}$$

Now consider the number Y_i of offspring of a single newborn individual when the population size is large. With probability β_i, the individual will have a Poisson number of offspring with mean and variance equal to $\frac{\chi_i}{\beta_i}$. Thus, the expected number of its offspring is equal to 1. By conditioning on the type of nest site an individual ends up occupying, we have

$$\sigma^2 = \sum_{i=1}^{L} \beta_i (\frac{\chi_i}{\beta_i} + (\frac{\chi_i}{\beta_i})^2) - 1 = \sum_{i=1}^{L} \frac{(\chi_i)^2}{\beta_i}. \qquad \text{[SI19]}$$

Comparing Equation (SI18) and (SI19), we see that $N_e = \frac{N}{\sigma^2}$ under this nest-site model, and since this is a Cannings model, Kingman's coalescent is the ancestral process in the limit as N goes to infinity and time is measured in units of N_e generation, provided that $0 < \sigma^2 < \infty$.

E. Proof of theorems related to the properties of *fractional* coalescent. In this section, we present the proof of theorems which have been presented on section 2B.

Theorem 1: Suppose $f_{T_i}(t) = t^{\alpha_i - 1}\lambda_i \mathcal{E}_{\alpha_i,\alpha_i}(-\lambda_i t^{\alpha_i})$ is the distribution of a waiting time in the f-coalescent where $T_i, \quad i = 2, ..., n$ are the coalescent times and $\lambda_i = \binom{i}{2}$ if $\alpha_1 = \alpha_2 = ... = \alpha_n$ then the distribution of time to the most recent common ancestor $T_{\mathbf{MRCA}} = \sum_{i=2}^{n} T_i$ is as follows

$$f_{T_{\mathbf{MRCA}}}(t) = \sum_{i=2}^{n} (\prod_{\substack{k=2 \\ k \neq n}}^{n} \frac{\lambda_k}{\lambda_k - \lambda_i}) f_{T_i}(t), \qquad \text{[SI20]}$$

or, equivalently, this can be presented as

$$f_{T_{\mathbf{MRCA}}}(t) = \sum_{i=2}^{n} (\frac{(2i-1)(-1)^i n_{[i]}}{n_{(i)}}) f_{T_i}(t), \qquad \text{[SI21]}$$

where

$$n_{[i]} = n(n-1)...(n-i+1), \;\; n_{(i)} = n(n+1)...(n+i-1).$$

Proof: If $T_{\text{MRCA}} = \sum_{i=2}^{n} T_i$ then its distribution is as follows

$$f_{T_{\text{MRCA}}}(t) = f_{\sum_{i=2}^{n} T_i}(t), \qquad \text{[SI22]}$$

using the Laplace transform we have

$$L[f_{T_{\text{MRCA}}}(t)] = L[f_{\sum_{i=2}^{n} T_i}(t)] = \prod_{i=2}^{n} L[f_{T_i}(t)]. \qquad \text{[SI23]}$$

Also using the property of Mittag-Leffler function (5) we know

$$\int_0^\infty e^{-st} t^{\alpha_i - 1} \mathcal{E}_{\alpha_i,\alpha_i}(-\lambda t^{\alpha_i}) dt = \frac{1}{s^{\alpha_i} + \lambda}, \qquad \text{[SI24]}$$

DRAFT

using Eqs. (SI23) and (SI24) we have

$$L[f_{T_{\mathrm{MRCA}}}(t)] = \prod_{i=2}^{n} \frac{\lambda_i}{s^{\alpha_i} + \lambda_i}. \qquad \text{[SI25]}$$

Assume $\alpha_1 = \alpha_2 = ... = \alpha_n = \alpha$, by using Eq. (SI25) and partial fraction expansion we have

$$L[f_{T_{\mathrm{MRCA}}}(t)] = (\prod_{i=2}^{n} \lambda_i) \sum_{j=2}^{n} \frac{1}{\prod_{\substack{k=2 \\ k\neq j}}^{n}(\lambda_k - \lambda_j)} \frac{1}{s^{\alpha} + \lambda_j}. \qquad \text{[SI26]}$$

Using Eq. (SI26) and Laplace inverse we have

$$f_{T_{\mathrm{MRCA}}}(t) = \sum_{i=2}^{n} (\prod_{\substack{k=2 \\ k\neq n}}^{n} \frac{\lambda_k}{\lambda_k - \lambda_i}) f_{T_i}(t). \qquad \text{[SI27]}$$

Also we know $(\prod_{\substack{k=2 \\ k\neq n}}^{n} \frac{\lambda_k}{\lambda_k - \lambda_i}) = (\frac{(2i-1)(-1)^i n_{[i]}}{n_{(i)}})$ (3) so by using Eqs. (SI26) and (SI27) we can derive Eq. (SI21).

Theorem 2: With the same assumption in Theorem 1, in f-coalescent, the probability $P_{nm}(T)$ that n genes are descendants from m genes T units of time ago is

$$P_{nm}(T) = \begin{cases} \sum_{i=m+1}^{n} (\prod_{\substack{k=m+1 \\ k\neq n}}^{n} \frac{\lambda_k}{\lambda_k - \lambda_i})(\frac{\lambda_i}{\lambda_m - \lambda_i}\mathcal{E}_{\alpha}(-\lambda_i T^{\alpha}) + \frac{\lambda_i}{\lambda_i - \lambda_m}\mathcal{E}_{\alpha}(-\lambda_m T^{\alpha})) & 1 < m < n \\ \sum_{i=2}^{n} (\prod_{\substack{k=2 \\ k\neq n}}^{n} \frac{\lambda_k}{\lambda_k - \lambda_i})(-\mathcal{E}_{\alpha}(-\lambda_i T^{\alpha}) + \mathcal{E}_{\alpha}(0)) & m = 1 \\ \mathcal{E}_{\alpha}(-\lambda_n T^{\alpha}) & m = n. \end{cases} \qquad \text{[SI28]}$$

Proof: If $s_{nm} = \sum_{i=m+1}^{n} T_i$ where T_i is the a coalescent time then the probability that n genes descended from m ancestral genes T units of time ago is as follows

$$P_{nm}(T) = P\{s_{nm} < T \leq s_{nm} + T_m\} = \int_0^T d\tau P\{s_{nm} = \tau\} \int_{T-\tau}^{\infty} f_{T_m}(t')dt'. \qquad \text{[SI29]}$$

Also we know

$$\int f_{T_n}(t')dt' = -\mathcal{E}_{\alpha}(\lambda_n t^{\alpha}), \qquad \text{[SI30]}$$

using Eqs. (SI20), (SI29) and (SI30) we have

$$P_{nm}(T) = \sum_{i=m+1}^{n} (\prod_{\substack{k=m+1 \\ k\neq n}}^{n} \frac{\lambda_k}{\lambda_k - \lambda_i}) \int_0^T \lambda_i \tau^{\alpha-1} \mathcal{E}_{\alpha,\alpha}(-\lambda_i \tau^{\alpha}) \times \mathcal{E}_{\alpha}(-\lambda_m (T-\tau)^{\alpha}) d\tau. \qquad \text{[SI31]}$$

Now we calculate the integral which is in Eq. (SI31). If

$$A = \int_0^T \lambda_i \tau^{\alpha-1} \mathcal{E}_{\alpha,\alpha}(-\lambda_i \tau^{\alpha}) \times \mathcal{E}_{\alpha}(-\lambda_m (T-\tau)^{\alpha}), \qquad \text{[SI32]}$$

using the Laplace transform and partial fractional expansion we get

$$L[A] = \frac{1}{s}\frac{\lambda_i}{\lambda_i - \lambda_m}\frac{1}{s^{\alpha} + \lambda_i} + \frac{1}{s}\frac{\lambda_m}{\lambda_m - \lambda_j}\frac{1}{s^{\alpha} + \lambda_m}. \qquad \text{[SI33]}$$

Since $\int \lambda_i T^{\alpha-1} \mathcal{E}_{\alpha,\alpha}(-\lambda_i T^{\alpha}) = -\mathcal{E}_{\alpha}(-\lambda_i T^{\alpha})$, using Eq. (SI33) and the Laplace inverse we have

$$A = \frac{\lambda_i}{\lambda_m - \lambda_i}(\mathcal{E}_{\alpha}(-\lambda_i T^{\alpha}) - \mathcal{E}_{\alpha}(-\lambda_m T^{\alpha})), \qquad \text{[SI34]}$$

using Eqs. (SI31) and (SI34) we can derive Eq. (SI28) for $1 < m < n$, and we calculate $P_{n1}(T)$ and $P_{nn}(T)$ as follows

$$P_{n1}(T) = P(s_{n1} \leq T) = \sum_{i=2}^{n} (\prod_{\substack{k=2 \\ k\neq n}}^{n} \frac{\lambda_k}{\lambda_k - \lambda_i}) \int_0^T f_{T_i}(\tau)d\tau = \sum_{i=2}^{n} (\prod_{\substack{k=2 \\ k\neq n}}^{n} \frac{\lambda_k}{\lambda_k - \lambda_i})(-\mathcal{E}_{\alpha}(-\lambda_i T^{\alpha}) - \mathcal{E}_{\alpha}(0)). \qquad \text{[SI35]}$$

DRAFT

Also we have

$$P_{nn}(T) = P(T \le T_n) = \int_T^\infty f_{T_n}(t')dt' = \mathcal{E}_\alpha(-\lambda_n T^\alpha). \qquad \text{[SI36]}$$

We have plotted Eq. (SI20) (or Eq. (SI21)) in Figure 1 for different values of α; α=1 is equivalent to the n-coalescent. For small value of α (α=0.3) the distributions are the same for all value of n. This result will be confirmed in the section J where we see the datasets generated with α<0.4 most commonly did not have any variable sites. Figure 1 shows, for 0.3<α<1, the mode of $f_{T_{\rm MRCA}}(t)$ is decreased when n is increased, and modes are about the same point for all value of n (The mode of $f_{T_{MRCA}}(t)$ moves very slowly to the right). For n-coalescent (α=1) the mode of $f_{T_{MRCA}}(t)$ is fixed but they are at different points for different values of n (The mode of $f_{T_{MRCA}}(t)$ moves fast to the right, but the value of mode does not change as n increase). The asymmetry of $f_{T_{MRCA}}(t)$ is stronger for α<1. While for α=1 as n increases, the distribution of $f_{T_{MRCA}}(t)$ converges on a distribution with mean equal 2 which corresponds to a period of $2N$ generations under the haploid Wright-Fisher model (3), for α<1 we have a heavy-tailed distributions as n increases.
Since for α<1, $E_\alpha(-\lambda_i T^\alpha)$ in Eq. (SI28) is greater than $e^{-\lambda_i T}$, in the f-coalescent as T increases m does not decrease as rapidly as in the n-coalescent (6).

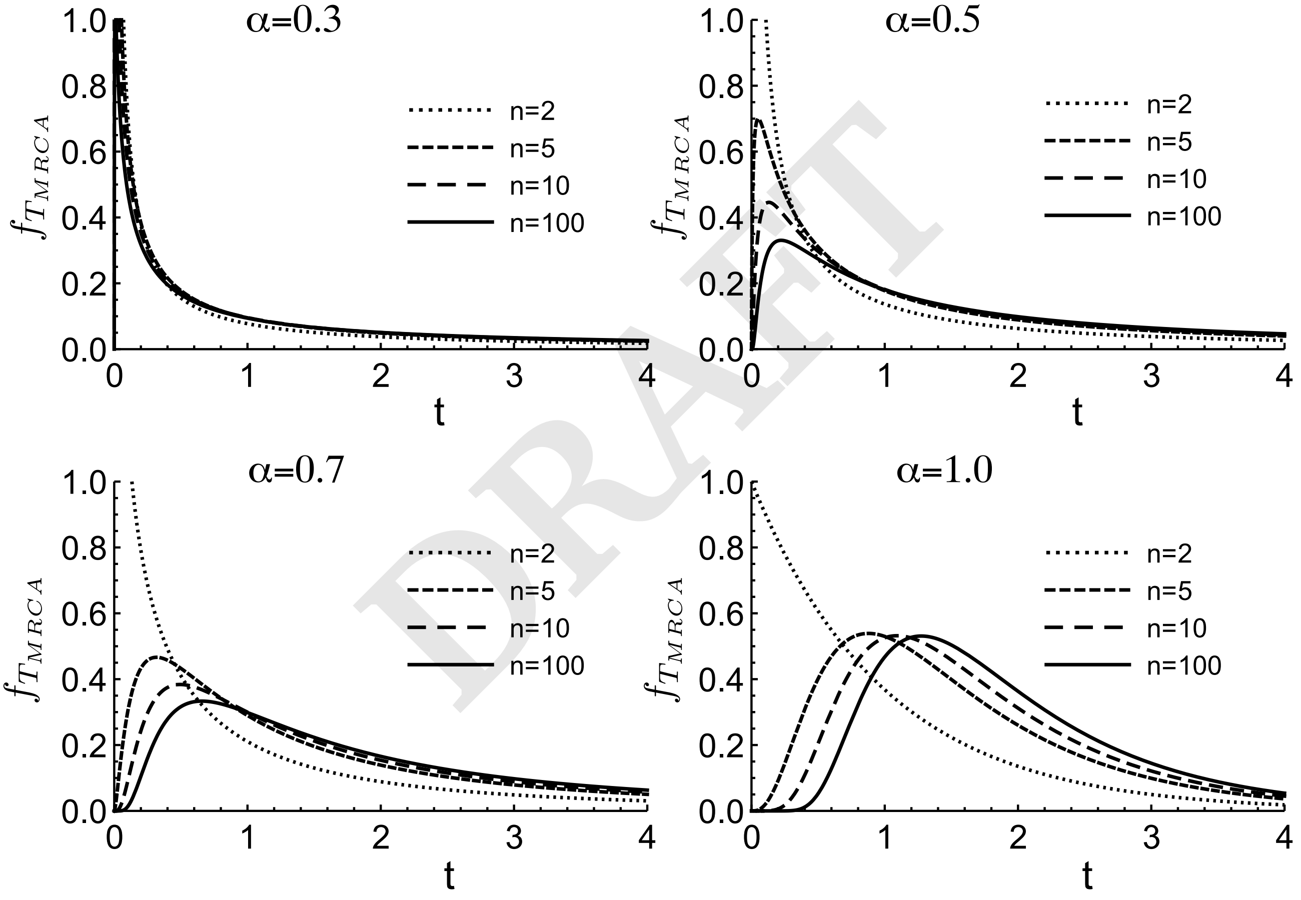

DRAFT

Fig. 1. Distribution of the time to the most recent common ancestor for different value of α and different samples sizes n.

F. Numerical values of the probability that *n* genes are descendants from *m* ancestral genes. Table 1 gives the numerical values of the probability that n genes sampled from a population are descendants from m ancestral genes T units of time ago ($P_{nm}(t)$ in Eq. (SI28)) for different value of α. It is noted that m decreases quite rapidly as T increases for the n-coalescent (α=1), but this not the case for f-coalescent. Similar results for the n-coalescent have been reported by Takahata (6).

G. Probability density function of a genealogy based on Kingman's *n*-coalescent. The waiting times in Kingman's n-coalescent have an exponential distribution and we express the probability density function of a time interval u_k with k lineages as

$$g_k(u_k|\Theta) = \lambda_k e^{-\lambda_k u_k}, \qquad \text{[SI37]}$$

$$\qquad \text{[SI38]}$$

where

$$\lambda_k = \frac{k(k-1)}{\Theta} \tag{SI39}$$

[SI40]

with the mutation-scaled population size $\Theta = 2N\mu$ where N is effective population size and μ is mutation rate per generation.

H. Deriving the probability density function of a genealogy based on the *fractional* coalescent. The probability density function of a genealogy based on the Kingman's n-coalescent is shown in G. For the f-coalescent we express the waiting time with the fractional generalization of the exponential probability distribution. The exponential function is replaced by the generalized Mittag-Leffler function $\mathcal{E}_{\alpha,\alpha}$ with the parameter α (N). Using Eq. (9), the probability that two of k lineages coalesce after one unit of scaled time is

$$\mathcal{E}_\alpha(-\binom{k}{2}\tau^\alpha). \tag{SI41}$$

When we replace the scaled time τ with

$$\frac{u_k}{(N\mu)^{\frac{1}{\alpha}}}, \tag{SI42}$$

where u_k has been scaled based on mutation ($\mu^{1/\alpha}$), we can rewrite Eq. (SI41) as

$$\mathcal{E}_\alpha(-\frac{k(k-1)}{2}\frac{u_k^\alpha}{N\mu}). \tag{SI43}$$

Replacing $2N\mu$ with Θ we get

$$\mathcal{E}_\alpha(-\frac{k(k-1)}{\Theta}u_k^\alpha). \tag{SI44}$$

Using Eq. (SI44) and (SI51), the probability density for a time interval u_k is

$$f_k(u_k|\Theta) = u_k^{\alpha-1}\lambda_k\mathcal{E}_{\alpha,\alpha}(-\lambda_k u_k^\alpha), \tag{SI45}$$

where λ_k is introduced in Eq. (SI39). We consider values for α in the interval $(0,1]$. The Mittag-Leffler function reduces to the exponential function with α=1 and Eq. (SI45) reduces to the familiar probability density for a time interval u_k for n-coalescent. The probability density of all observed u_k with $k = 2, ..., K$ given the mutation-scaled population size Θ is the joint probability distribution function

$$f(u_2, u_3, ..., u_K|\Theta) = \prod_{k=2}^{K} f_k(u_k|\Theta) = \prod_{k=2}^{K} u_k^{\alpha-1}\lambda_k\mathcal{E}_{\alpha,\alpha}(-\lambda_k u_k^\alpha), \tag{SI46}$$

where K is the number of samples. To extract a particular genealogy G out of the many possible topologies defined by the interval times $u_2, u_3, ..., u_K$ we use Eq. (15).

I. Implementing the Mittag-Leffler function. A particular problem for a useful implementation was the calculation time of the generalized Mittag-Leffler function. We programmed the function in the C-language using the algorithm described by Gorenflo (7) that also was used to create a Matlab-function by I. Podlubny (https://www.mathworks.com/matlabcentral/fileexchange/8738-mittag-leffler-function). We compared our C Mittag-Leffler function with the one implemented in Mathematica 11.0.1.0 (8) for correctness. During these tests we realized that our implementation will be very slow in a MCMC run, because the function will be called millions of times. We implemented a lookup table for values of the Mittag-Leffler functions $\mathcal{E}_\alpha(x)$ and $\mathcal{E}_{\alpha,\alpha}(x)$ for $\alpha \in [0.01, 0.02, 0.03, ...0.99]$. The lookup-table values were created by finding all values for a given α that have $d\mathcal{E}_{\alpha,\alpha}(x)/dx \leq 0.01$ and $d\mathcal{E}_{\alpha,1}(x)/dx \leq 0.01$. This lookup-table is then used with a Hermite-cubic-interpolation and a linear interpolation at the table end points to return values for the Mittag-Leffler functions. This interpolation is roughly 2x faster than our original C-function. Our lookup table and interpolation function can be downloaded from http://popgen.sc.fsu.edu/codes.

J. Methods. *Time to the most recent common ancestor for the f-coalescent:* The most recent common ancestor of the sampled individuals is at the root of a genealogy. The time of this most recent common ancestor (T_{MRCA}) is directly related to the effective population size of the population from which the sample was taken. Coalescence theory assumes that each time interval j is independent from the time interval $j-1$ or $j+1$. If we are only interested in the population size, we only need to consider the time intervals and do not need to know details of the topology of the genealogy. We compared empirical distributions of the T_{MRCA} for a sample of 5 individuals for the f-coalescent, n-coalescent, and Bolthausen-Sznitman-coalescent (BS-coalescent; 9). BS-coalescent allows merging of more than two lineages and is used to describe the waiting times in a genealogy that is affected by selection (10). All calculations were executed in Mathematica 11.0.1 (8). For the n-coalescent, BS-coalescent, and the f-coalescent we summed independently drawn time intervals for 5, 4, 3, and 2 lineages to get the T_{MRCA}.

DRAFT

We drew 100,000 replicated T_{MRCA} for each of the three n-coalescent settings (strong population shrinkage, fixed population size, strong population growth), two BS-coalescent settings ($T_c = 0.005$ and 0.01) and the two f-coalescent settings ($\alpha = 0.9$ and 0.8). For the fixed population size n-coalescent time intervals, we solved Eq. (3) in (11); for time intervals of the growing and shrinking population we used Eq. (4.6) in (12); for the BS-coalescent we used the algorithm of (10); for the f-coalescent we used Eq. (17). Comparison between the f-coalescent and the BS-coalescent are more difficult because of the parametrization. Neher (10) postulates that the coalescent rates of the BS-coalescent and the n-coalescent are not equivalent, the parameter T_c in BS-coalescent defines the time to the selectively positive innovation whereas in the f-coalescent and the n-coalescent the rate is defined by the scaled population size. There is certainly a direct relationship between T_c and Θ but under selection that relationship changes.

Simulated data: We evaluated the algorithms using simulations. We updated our simulator package (available at peter-beerli.com/software and bitbucket.com) to allow generating genealogies from the f-coalescent. We generated 100-locus datasets with a population size $N_e = 2500$ and mutation rate per site per generation $\mu = 0.000001$ for α in $[0.4, 0.5, ..1.0]$. Each locus had 10,000 base pairs. Datasets generated with $\alpha < 0.4$ most commonly did not have any variable sites. The datasets were then analyzed with MIGRATE with a fixed α of 0.4 or $0.5, ...,$ or 1.0. Each dataset was thus analyzed 7 times with different α values allowing us to compare the different runs considering each α value as a different model. We use the framework of marginal likelihoods (cf. 13) to compare the different models. The method implemented in MIGRATE uses thermodynamic integration to approximate the marginal likelihoods (14, 15). We then used these marginal likelihood as model weights (16) to decide whether models could be considered to be appropriate for the data or not. The results (Figure 2) show that exact estimation of α from a single locus is difficult.

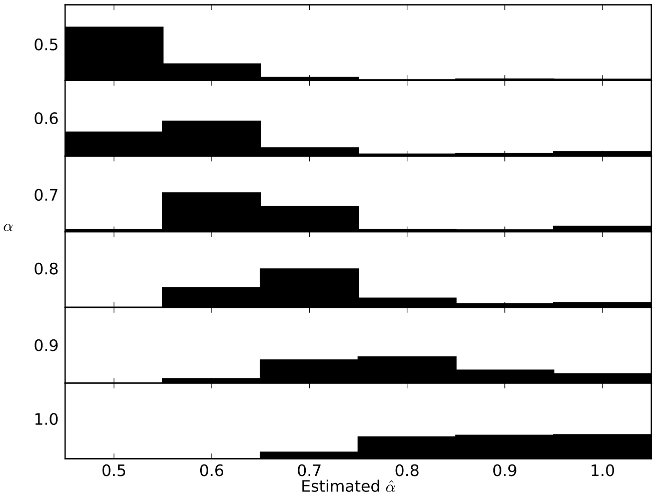

Fig. 2. Comparison of recovery of the model under which the data was simulated. α was used to simulate the data; The histograms of $\hat{\alpha}$ show the number of wins in a marginal likelihood comparison. For each true α, 500 simulations with 10 individuals and 10,000 base pairs were used.

The f-coalescent has an additional parameter α which could reflect hidden structure. To explore whether the parameter α responds to population structure. We simulated data from two subpopulations (20 individuals each) for 10 loci (10,000 bp each) using mutation-scaled population size $\Theta_1 = \Theta_2 = 0.01$ and symmetric immigration rates $M_{1\to 2} = M_{3\to 1} = 1$, 100, and $10,000$. Θ is $4\times$ the effective population size N_e $\times$ the mutation rate μ per site and generation; M is the immigration rate scaled by the mutation rate. The scaled immigration rates are equivalent to the more familiar $4N_em$ of $0.01, 1.0,$ and 100. We then ran several models: (1) the correct structured n-coalescent model with immigration and two subpopulation sizes; (2) panmictic model using the n-coalescent, (3) panmictic model using the f-coalescent with $\alpha < 1$. We ran models for $\alpha = 0.5, 0.06, 0.7, 0.8,$ and 0.9 (All simulation files are available on `https://github.com/pbeerli/fractional_coalescent_data`). We compared the different models using Bayesian model comparison (14, 15). We reanalyzed this same data focusing only on population 1 assuming that we did not recognize population 2, using single population models for the n-coalescent and the f-coalescent, and a ghost-population model (17) that assumes we know about a second population but do not have any data for it.

Real data: We used three biological datasets to explore whether the f-coalescent could be a better fit than Kingman's n-coalescent. The first dataset is a dataset of short mitochondrial control region sequences of the humpback whale population in the North Atlantic (18), the second is a small dataset of the malaria parasite *Plasmodium falciparum* in Africa (19), and the third dataset is a small dataset of the H1N1 influenza strain that scared the world in 2014 with an outbreak in Mexico (downloaded on August 7 2017 from www.fludb.org). We used a simple mutation model, F84 + G (20, 21), optimized the transition/transversion ratio (ts/tv) and the site rate parameters in PAUP* (22). For the whale and Plasmodium data the ts/tv were 18.08 and 1.56, respectively. The site rate variation parameter estimates were 0.0277 and 0.00982. The influenza data were heterogeneous: segment 1-4 had a site rate variation parameter of ~ 0.02, and the segments 5-8 had a value of

> 200.0. We analyzed the total influenza data as 8 independent loci using the F84 model. All data were then run using $\alpha = [0.4, 0.5, ..., 1.0]$. The influenza data was also analyzed using a exponential growth n-coalescent model. All models were then compared using marginal likelihoods.

K. Using the *fractional* coalescent for structured coalescent models. The fractional coalescent introduces a parameter that is linked to the variance of waiting times between events. The standard Kingman's n-coalescent has exponentially distributed waiting times that depend on the rate of coalescent and the number of lineages at risk to coalesce. The f-coalescent uses the same rate of coalescence and lineages, but allows for further variability; with a low α the variability is high and with $\alpha = 1$ the variability is equivalent to the n-coalescent. When the population is structured the immigration process can also introduce variability of waiting times between coalescent events. Here we evaluate whether this variability influences α, so that α could be interpreted as an indicator of missed population structure.

This may lead to intervals between coalescent times that cannot be explained by the n-coalescent and would potentially allow a process that can accommodate more variable waiting times such as the f-coalescent to better fit the data. The model comparison of the structured datasets rejected any model that uses the f-coalescent when using the data from both of the simulated locations. The structured n-coalescent was preferred (Table 2) for cases when the data was simulated with low or medium magnitude of gene flow; data simulated with high gene flow is indistinguishable from a panmictic population, thus the single-population model was preferred. When analyzing the data using only a one of the two populations (Table 3), with large gene flow the n-coalescent model is preferred; with low immigration the f-coalescent model with an $\alpha = 0.9$ is preferred over all other models. Rare immigration will introduce new genetic material into a population which will lead to a waiting time between recorded coalescent events that has very low probability in the n-coalescent framework but is normal in the f-coalescent framework that allows higher variability of the waiting times.

We can conclude that the single-population f-coalescent is a poor model when the immigration is not very low and when we are aware that there are more populations (cf. 17). This manuscript discusses the single population f-coalescent and we intend to improve on the theory to include multiple populations and include forces such as ongoing immigration and population divergence.

DRAFT

L. The Fractional Poisson Process. The classical Poisson process is a common random process in probability theory. Several probability distributions arise from the classical Poisson process, for example the Poisson distribution and the exponential distribution. In this process, the number of events, $N(u)$, which happen during the time interval from 0 to u has a Poisson distribution; and, the waiting times between these events are exponentially distributed. Kingman's n-coalescent uses the classical Poisson process to model the waiting time between two coalescent events. For the f-coalescent, we change the distribution of waiting times from an exponential distribution to the fractional generalization of the exponential probability distribution.

Before we introduce the fractional generalization of the exponential probability distribution, we first introduce the fractional Poisson Process.

Fractional Poisson processes have been developed by using the classical Poisson process in two different cases, the time-fractional Poisson process and space-fractional Poisson process (23). The combination of these two different cases has been introduced as the space-time-fractional Poisson process (23).

For the classical homogeneous Poisson process we know that

$$p_k(u) = Pr\{N(u) = k\} = e^{-\lambda u}\frac{(\lambda u)^k}{k!}, \qquad u \geq 0,\ \lambda > 0,\ k \geq 0, \qquad \text{[SI47]}$$

it is also known that $p_k(u)$ with $k{\geq}0$ solves the following Cauchy problem

$$\frac{d}{du}p_k(u) = -\lambda p_k(u) + \lambda p_{k-1}(u), p_k(0) = \delta_{k0}, \qquad \text{[SI48]}$$

where $\delta_{n,0}$ is the Kronecker symbol. Time-fractional generalizations of the Poisson process are based on the substitution of the integer-order derivative operator appearing in Eq. (SI48) with a fractional-order derivative operator such as the Riemann-Liouville fractional derivative (24) or the Caputo fractional derivative (25) (see M). Properties of the time-fractional Poisson process have been studied in (26) and (27). Space-fractional generalizations of the Poisson process are based on the substitution of the backward shift operator space in Eq. (SI48) with the fractional backward shift operator (23).

We use the time-fractional generalization of the homogeneous Poisson process which has been introduced in (24). In this generalization, the fractional Poisson process has been introduced as the counting process with probability $P_\alpha(n, u)$ of having n items $(n = 0, 1, 2, ...)$ during time interval u by the following special form of the fractional Kolmogorov-Feller equation for $0 < \alpha \leq 1$

$$D_R^\alpha P_\alpha(n, u) = \lambda(P_\alpha(n-1, u) - P_\alpha(n, u)) + \frac{u^{-\alpha}}{\Gamma(1-\alpha)}\delta_{n,0}, \qquad \text{[SI49]}$$

where $\delta_{n,0}$ is the Kronecker symbol with normalization condition

$$\sum_{n=0}^{\infty} P_\alpha(n, u) = 1,$$

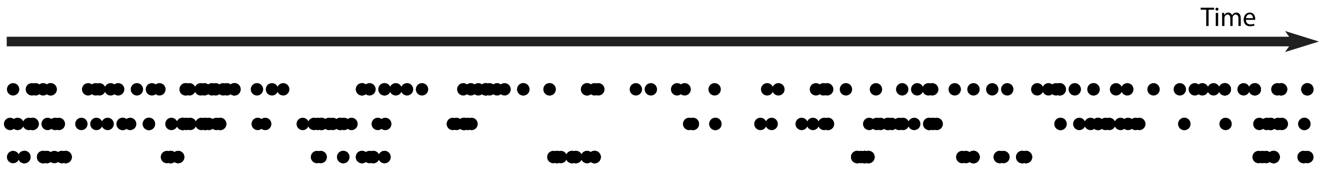

Fig. 3. Times between events drawn using the classical Poisson process and the fractional Poisson process with $\alpha = 0.9$, and 0.8; from top to bottom.

By solving Eq. (SI49) we have (24)

$$P_\alpha(n,u) = \frac{(\lambda u^\alpha)^n}{n!}\sum_{k=0}^{\infty}\frac{(k+n)!}{k!}\frac{(-\lambda u^\alpha)^k}{\Gamma(\alpha(k+n)+1)}, \quad 0<\alpha\le 1. \qquad \text{[SI50]}$$

In this generalization, the probability distribution function of waiting time for the fractional Poisson process can be expressed as

$$\psi_\alpha(u) = -\frac{d}{du}P_\alpha(u), \qquad \text{[SI51]}$$

where $P_\alpha(u)$ is the probability that a given inter arrival time is greater or equal to u

$$P_\alpha(u) = 1-\sum_{n=1}^{\infty}P_\alpha(n,u) = \mathcal{E}_\alpha(-\lambda u^\alpha), \qquad \text{[SI52]}$$

where $\mathcal{E}_\alpha$ is the Mittag-Leffler function see (N). By using Eqs. (SI51) and (SI52) the waiting time for fractional Poisson process has the following probability distribution function

$$\psi_\alpha(u) = \lambda u^{\alpha-1}\mathcal{E}_{\alpha,\alpha}(-\lambda u^\alpha), \quad u\ge 0, \quad 0<\alpha\le 1, \qquad \text{[SI53]}$$

where $\mathcal{E}_{\alpha,\alpha}$ is the generalization of the Mittag-Leffler function (see N). The $\psi_\alpha(u)$ defined by Eq. (SI53) is fractional generalization of the well known exponential probability distribution.
The generalization of the Mittag-Leffler function has two different parameters α and β (see N), and as it has been mentioned in Eq. (SI53) the fractional Poisson process, has been derived by using the Mittag-Leffler function using ($\alpha = \beta$). Figure 3 shows the time of 100 independent events which happen in three cases of the Poisson process with various α (1.0, 0.9, and 0.8). For calculating these times we have supposed that the probability each event occurs is a random number while λ has been chosen to be fixed and same in all three different cases. With an $\alpha < 1$ more times became shorter but a few times become longer; and the variance of the times becomes larger.

M. Definitions of Fractional Derivatives. The various definitions for fractional derivatives exist. A commonly used definition is the Riemann-Liouville fractional derivative (28).
Definition : The Riemann-Liouville's fractional derivative of order α is defined as

$$(D_R^\alpha f)(t) = \frac{1}{\Gamma(n-\alpha)}\frac{d^n}{dt^n}\int_0^t \frac{f(s)}{(t-s)^{\alpha+1-n}}ds, \quad n-1<\alpha\le n, \qquad \text{[SI54]}$$

where $\alpha > 0$ is the order of the derivative and $n \in \mathbb{N}$ is the smallest integer greater than α. For the Riemann-Liouville's derivative we have

$$D_R^\alpha c = c\frac{t^{-\alpha}}{\Gamma(1-\alpha)}, \qquad \text{[SI55]}$$

where c is a constant. Replacing c with t^ν we get

$$D_R^\alpha t^\nu = \tfrac{\Gamma(\nu+1)}{\Gamma(\nu+1-\alpha)}t^{\nu-\alpha}, \quad n-1<\alpha<n,\ \nu>-1,\ \nu\in\mathbb{R}. \qquad \text{[SI56]}$$

DRAFT

N. The Mittag-Leffler Functions. The Mittag-Leffler functions $\mathcal{E}_\alpha(x)$ with $\alpha > 0$ have been introduced as (29)

$$\mathcal{E}_\alpha(x) = \sum_{n=0}^{\infty} \frac{x^n}{\Gamma(\alpha n + 1)}, \quad \alpha > 0, x \in \mathcal{C}. \qquad \text{[SI57]}$$

This function provides a simple generalization of the exponential function because of the substitution in the exponential series of $n! = \Gamma(n+1)$ with $(\alpha n)! = \Gamma(\alpha n + 1)$. A straightforward generalization of the Mittag-Leffler function is obtained by replacing the additive constant 1 in the argument of the *Gamma* function in Eq. (SI57) by an arbitrary complex parameter β. For this function we use the following notation

$$\mathcal{E}_{\alpha,\beta}(x) = \sum_{n=0}^{\infty} \frac{x^n}{\Gamma(\alpha n + \beta)}, \quad \alpha > 0, \beta, x \in \mathcal{C} \qquad \text{[SI58]}$$

of course $\mathcal{E}_{\alpha,1}(x) = \mathcal{E}_\alpha(x)$. The Mittag-Leffler function can be expressed as a mixture of exponentials (30)

$$\mathcal{E}_\alpha(-\lambda x^\alpha) = \int_0^\infty \omega(\kappa, \alpha) e^{-\kappa x} d\kappa, \qquad \text{[SI59]}$$

where $\omega(\kappa, \alpha)$ is the probability density

$$\omega(\kappa, \alpha) = \lambda \frac{sin(\alpha\pi)}{\pi} \frac{\kappa^{\alpha-1}}{\kappa^{2\alpha} + 2\lambda\kappa^\alpha cos(\alpha\pi) + \lambda^2}. \qquad \text{[SI60]}$$

The discrete form of Eq. (SI59) is

$$\mathcal{E}_\alpha(-\lambda x^\alpha) = \sum_k \omega(\kappa_k, \alpha)(1 - \kappa_k \frac{x}{n})^n, \qquad \text{[SI61]}$$

as $k \to \infty$. If $n \to \infty$ then $\sum_k \omega(\kappa_k, \alpha)(1 - \kappa_k \frac{x}{n})^n \to \int_0^\infty \omega(\kappa, \alpha) e^{-\kappa x} d\kappa$. It should be noted since summation in Eq. (SI61) is converging as $n \to \infty$, we can rewrite Eq. (SI61) as

$$\mathcal{E}_\alpha(-\lambda x^\alpha) = \sum_k \omega(\kappa_k, \alpha)(1 - \kappa_k \frac{1}{n})^{xn}. \qquad \text{[SI62]}$$

A Mittag-Leffler matrix function (30) can be introduced as continuous form

$$\mathcal{E}_\alpha(AT^\alpha) = \int_0^\infty \omega(s, \alpha) exp(-s(-A)^{\frac{1}{\alpha}} t) ds, \qquad \text{[SI63]}$$

where $\lambda = 1$ and a discrete form

$$\mathcal{E}_\alpha(At^\alpha) = \sum_k \omega(\kappa_k, \alpha)(I + \frac{-\kappa_k(-A)^{\frac{1}{\alpha}} t}{n})^n, \qquad \text{[SI64]}$$

as $k \to \infty$ where $A \in R^{n \times n}$ and $\lambda = 1$.

O. Notes. In this section, we clarify how in Eq. (3), in the main text, the probability mass function of χ_i depends on the probability mass function of σ^2. This dependency is governed by following proposition.

Proposition: Let X be a discrete random variable with support R_X and probability mass function $p_X(x)$. Let $g : R \to R$ be strictly increasing on the support of X. Then, the support of $Y = g(X)$ is

$$R_Y = \{y = g(x) : x \in R_X\}$$

and its probability mass function is (31)

$$p_Y(y) = \begin{cases} p_X(g^{-1}(y)) & \textbf{if} \quad y \in R_Y \\ 0 & \textbf{if} \quad y \notin R_Y \end{cases}. \qquad \text{[SI65]}$$

Suppose $\chi_i,\ i = 1, ..., L$ are discrete random variables and the probability mass functions of $\chi_i,\ i = 1, ..., L$ are $p_{\chi_i}(x),\ i = 1, ..., L$. We introduce discrete random variables $Y_i,\ i = 1, ..., L$ as

$$Y_i = \frac{(\chi_i)^2}{\beta_i}. \qquad \text{[SI66]}$$

DRAFT

Using Eqs. (SI65) and (SI66) the probability mass functions of $Y_i,\ i = 1, ..., L$ is as

$$p_{Y_i}(y_i) = \begin{cases} p_{\chi_i}(\sqrt{y_i \beta_i}) & \text{if } y_i \in R_{Y_i} \\ 0 & \text{if } y_i \notin R_{Y_i} \end{cases}. \qquad \text{[SI67]}$$

On the other hand the summation of the discrete random variables $Y_i,\ i = 1, ..., L$ is as

$$\sigma^2 = \sum_{i=1}^{L} Y_i = \sum_{i=1}^{L} \frac{(\chi_i)^2}{\beta_i}, \qquad \text{[SI68]}$$

which has the probability mass functions equal $\omega(\sigma^2, \alpha)$. This shows the probability mass functions of $Y_i,\ i = 1, ..., L$ $(\chi_i,\ i = 1, ..., L)$ should satisfy both Eqs. (SI67) and (SI68).

1. Kingman JF (1982) On the genealogy of large populations. *Journal of Applied Probability* 19(A):27–43.
2. Nordborg M (2001) Coalescent theory. *Handbook of statistical genetics* 2:843–877.
3. Wakeley J (2009) *Coalescent theory: an Introduction.* (Roberts & Company Publishers, Greenwood Village, Colorado. pp 326).
4. Der R, Epstein CL, Plotkin JB (2011) Generalized population models and the nature of genetic drift. *Theoretical population biology* 80(2):80–99.
5. Haubold HJ, Mathai AM, Saxena RK (2011) Mittag-Leffler functions and their applications. *Journal of Applied Mathematics* 2011:1–51.
6. Takahata N, Nei M (1985) Gene genealogy and variance of interpopulational nucleotide differences. *Genetics* 110(2):325–344.
7. Gorenflo R, Loutchko J, Luchko Y (2002) Computation of the Mittag-Leffler function $e_{\alpha,\beta}(z)$ and its derivative. *Fractional Calculus and Applied Analysis* 5(4):491–518.
8. Wolfram Research (2017) Mathematica, Version 11.1. Champaign, IL.
9. Bolthausen E, Sznitman AS (1998) On Ruelle's probability cascades and abstract cavity method. *Communications in Mathematical Physics* 197(2):247–276.
10. Neher RA, Hallatschek O (2012) Genealogies of rapidly adapting populations. *Proceedings of the National Academy of Sciences* 110(2):437–442.
11. Beerli P, Felsenstein J (1999) Maximum-likelihood estimation of migration rates and effective population numbers in two populations using a coalescent approach. *Genetics* 152(2):763–773.
12. Hein J, Schierup MH, Wiuf C (2005) *Gene Genealogies, Variation and Evolution: A Primer in Coalescent Theory.* (Oxford University Press, Oxford).
13. Kass RE, Raftery AE (1995) Bayes factors. *Journal of the American Statistical Association* 90(430):773–795.
14. Beerli P, Palczewski M (2010) Unified framework to evaluate panmixia and migration direction among multiple sampling locations. *Genetics* 185:313–326.
15. Palczewski M, Beerli P (2014) Population model comparison using multi-locus datasets in *Bayesian Phylogenetics: Methods, Algorithms, and Applications*, eds. Chen MH, Kuo L, Lewis PO. (CRC Press), pp. 187–200.
16. Burnham K, Anderson D (2002) *Model Selection and Multimodel Inference: A Practical Information-theoretic Approach.* (Springer, New York).
17. Beerli P (2004) Effect of unsampled populations on the estimation of population sizes and migration rates between sampled populations. *Molecular Ecology* 13(4):827–836.
18. Roman J, Palumbi S (2003) Whales before whaling in the North Atlantic. *Science* 301:508–510.
19. Joy DA, et al. (2003) Early origin and recent expansion of Plasmodium falciparum. *Science (New York, N.Y.)* 300(5617):318–321.
20. Felsenstein J, Churchill GA (1996) A hidden Markov model approach to variation among sites in rate of evolution. *Molecular Biology and Evolution* 13:93–104.
21. Yang Z (1993) Maximum-likelihood estimation of phylogeny from DNA sequences when substitution rates differ over sites. *Molecular Biology and Evolution* 10(6):1396–1401.
22. Swofford D (2003) PAUP*. Phylogenetic Analysis Using Parsimony (*and Other Methods). Version 4.
23. Orsingher E, Polito F (2012) The space-fractional Poisson process. *Statistics & Probability Letters* 82(4):852–858.
24. Laskin N (2003) Fractional Poisson process. *Communications in Nonlinear Science and Numerical Simulation* 8(3):201–213.
25. Beghin L, Orsingher E, , et al. (2009) Fractional Poisson processes and related planar random motions. *Electron. J. Probab* 14(61):1790–1826.
26. Meerschaert MM, Nane E, Vellaisamy P, , et al. (2011) The fractional Poisson process and the inverse stable subordinator. *Electronic Journal of Probability* 16(59):1600–1620.
27. Politi M, Kaizoji T, Scalas E (2011) Full characterization of the fractional Poisson process. *EPL (Europhysics Letters)* 96(2):20004.
28. Podlubny I (1998) *Fractional differential equations: an introduction to fractional derivatives, fractional differential equations, to methods of their solution and some of their applications.* (Academic press) Vol. 198.
29. Mainardi F (2010) *Fractional calculus and waves in linear viscoelasticity: an introduction to mathematical models.* (Imperial College Press).
30. MacNamara S, Henry B, McLean W (2017) Fractional Euler limits and their applications. *SIAM Journal on Applied Mathematics* 77(2):447–469.
31. Feller W (1968) *An introduction to probability theory and its applications.* (Wiley New York) Vol. 1.

DRAFT

Table 1. Probability values for n genes that are descendants from m ancestral genes after time T using the f-coalescent with different α values.

$\alpha = 0.3$

T	n	m				
		1	2	3	4	5
0.5	2	0.4895	0.5104			
	5	0.2723	0.3074	0.2020	0.1299	0.0000
	10	0.2176	0.2529	0.1697	0.1105	0.0756
1.0	2	0.5434	0.4565			
	5	0.3363	0.3029	0.1785	0.1095	0.0000
	10	0.2811	0.2596	0.1554	0.0962	0.0641
1.5	2	0.5745	0.4254			
	5	0.3753	0.2963	0.1648	0.0987	0.0000
	10	0.3207	0.2590	0.1460	0.0882	0.0580
2.0	2	0.5963	0.4036			
	5	0.4033	0.2900	0.1553	0.0916	0.0000
	10	0.3494	0.2566	0.1391	0.0826	0.0540

$\alpha = 0.5$

T	n	m				
		1	2	3	4	5
0.5	2	0.4768	0.5231			
	5	0.2417	0.3370	0.2158	0.1263	0.0000
	10	0.1818	0.2763	0.1910	0.1174	0.0756
1.0	2	0.5724	0.4275			
	5	0.3581	0.3262	0.1678	0.0915	0.0000
	10	0.2973	0.2901	0.1571	0.0881	0.0549
1.5	2	0.6268	0.3731			
	5	0.4309	0.3057	0.1419	0.0754	0.0000
	10	0.3727	0.2809	0.1357	0.0735	0.0452
2.0	2	0.6637	0.3362			
	5	0.4824	0.2868	0.1252	0.06560	0.0000
	10	0.4273	0.2683	0.1210	0.0643	0.0393

$\alpha = 0.7$

T	n	m				
		1	2	3	4	5
0.5	2	0.4541	0.5458			
	5	0.1894	0.3812	0.2485	0.1191	0.0000
	10	0.1230	0.3003	0.2408	0.1343	0.0749
1.0	2	0.6003	0.3996			
	5	0.3687	0.3721	0.1551	0.0677	0.0000
	10	0.2972	0.3469	0.1643	0.0761	0.0416
1.5	2	0.6830	0.3169			
	5	0.4880	0.3241	0.1122	0.0488	0.0000
	10	0.4239	0.3179	0.1200	0.0539	0.0298
2.0	2	0.7368	0.2631			
	5	0.5706	0.2803	0.08849	0.0389	0.0000
	10	0.5143	0.2812	0.0945	0.0424	0.0237

$\alpha = 1.0$

T	n	m				
		1	2	3	4	5
0.5	2	0.3934	0.6065			
	5	0.0812	0.4013	0.4030	0.1076	0.0000
	10	0.0248	0.2047	0.4051	0.2788	0.0770
1.0	2	0.6321	0.3678			
	5	0.3341	0.5297	0.1299	0.0060	0.0000
	10	0.2277	0.5256	0.2221	0.0237	0.0007
1.5	2	0.7768	0.2231			
	5	0.5695	0.3990	0.0311	0.0003	0.0000
	10	0.4823	0.4583	0.0580	0.0012	0.0000
2.0	2	0.8646	0.1353			
	5	0.7328	0.2600	0.0070	0.0000	0.0000
	10	0.6745	0.3119	0.0133	0.0000	0.0000

DRAFT

Table 2. Model comparison of datasets simulated under the two-population symmetric structured coalescent with 4 parameters ($\theta 1, \Theta_2, M_{2\to 1}$, and $M_{1\to 2}$) analyzed with different models using the data from both locations.

(a)

Simulated parameter	Model	$\ln mL$	LBF	Model probability	Rank
$\Theta_i = 0.01, M_{j\to i} = 1$	structured n-coalescent	-315237.51	0.00	1.00	1
	n-coalescent	-347952.07	-32714.56	0.00	2
	f-coalescent $\alpha = 0.9$	-329491.05	-14253.54	0.00	3
	f-coalescent $\alpha = 0.8$	-334463.19	-19225.68	0.00	4
	f-coalescent $\alpha = 0.7$	-339960.28	-24722.77	0.00	5
	f-coalescent $\alpha = 0.6$	-347409.42	-32171.91	0.00	6
	f-coalescent $\alpha = 0.5$	-356376.35	-41138.84	0.00	7
$\Theta_i = 0.01, M_{j\to i} = 100$	structured n-coalescent	-200371.94	0.00	1.00	1
	n-coalescent	-200591.59	-219.65	0.00	2
	f-coalescent $\alpha = 0.9$	-200891.26	-519.32	0.00	3
	f-coalescent $\alpha = 0.8$	-201371.88	-999.94	0.00	4
	f-coalescent $\alpha = 0.7$	-202014.66	-1642.72	0.00	5
	f-coalescent $\alpha = 0.6$	-202922.34	-2550.40	0.00	6
	f-coalescent $\alpha = 0.5$	-204198.38	-3826.44	0.00	7
$\Theta_i = 0.01, M_{j\to i} = 10000$	structured n-coalescent	-194729.51	-46.41	0.00	2
	n-coalescent	-194683.10	0.00	1.00	1
	f-coalescent $\alpha = 0.9$	-194898.44	-215.34	0.00	3
	f-coalescent $\alpha = 0.8$	-195213.06	-529.96	0.00	4
	f-coalescent $\alpha = 0.7$	-195735.97	-1052.87	0.00	5
	f-coalescent $\alpha = 0.6$	-196419.60	-1736.50	0.00	6
	f-coalescent $\alpha = 0.5$	-197584.96	-2901.86	0.00	7

DRAFT

Table 3. Model comparison of datasets simulated under the two-population symmetric structured coalescent with 4 parameters ($\theta 1, \Theta_2, M_{2\to 1}$, and $M_{1\to 2}$) analyzed with different models using the data from the first location.

Simulated parameter	Model	$\ln mL$	LBF	Model probability	Rank
$\Theta_i = 0.01, M_{j\to i} = 1$	structured n-coalescent	-162693.58	-236.50	0.0000	6
	n-coalescent	-162493.26	-36.18	0.00	3
	f-coalescent $\alpha = 0.9$	-162457.08	0.00	1.0	1
	f-coalescent $\alpha = 0.8$	-162477.44	-20.36	0.00	2
	f-coalescent $\alpha = 0.7$	-162551.20	-94.12	0.00	4
	f-coalescent $\alpha = 0.6$	-162682.19	-225.11	0.00	5
	f-coalescent $\alpha = 0.5$	-162864.37	-407.29	0.00	7
$\Theta_i = 0.01, M_{j\to i} = 100$	structured n-coalescent	-176290.61	-146.35	0.0000	3
	n-coalescent	-176144.26	0.00	1.00	1
	f-coalescent $\alpha = 0.9$	-176193.29	-49.03	0.00	2
	f-coalescent $\alpha = 0.8$	-176294.47	-150.21	0.00	4
	f-coalescent $\alpha = 0.7$	-176461.70	-317.44	0.00	5
	f-coalescent $\alpha = 0.6$	-176727.94	-583.68	0.00	6
	f-coalescent $\alpha = 0.5$	-177088.54	-944.28	0.00	7
$\Theta_i = 0.01, M_{j\to i} = 10000$	structured n-coalescent	-177937.84	-155.65	0.00	4
	n-coalescent	-177782.19	0.00	1.00	1
	f-coalescent $\alpha = 0.9$	-177814.14	-31.95	0.00	2
	f-coalescent $\alpha = 0.8$	-177910.57	-128.38	0.00	3
	f-coalescent $\alpha = 0.7$	-178052.81	-270.62	0.00	5
	f-coalescent $\alpha = 0.6$	-178291.38	-509.19	0.00	6
	f-coalescent $\alpha = 0.5$	-178614.13	-831.94	0.00	7
